# Dry eye symptom questionnaires show adequate measurement precision and psychometric validity for clinical assessment of vision-related quality of life in glaucoma patients

**DOI:** 10.1371/journal.pone.0283597

**Published:** 2023-03-24

**Authors:** Stephen Ocansey, Ebenezer Oduro Antiri, Carl Haladay Abraham, Emmanuel Kwasi Abu

**Affiliations:** 1 Department of Optometry and Vision Science, School of Allied Health Sciences, College of Health and Allied Sciences, University of Cape Coast, Cape Coast, Ghana; 2 Klintaps College of Health and Allied Sciences, Tema, Ghana; University of New South Wales, AUSTRALIA

## Abstract

**Purpose:**

To ascertain the presence of Dry Eye Syndrome (DES) in patients being treated for glaucoma, using subjective and objective methods and to examine DES impact on their quality of life (QOL).

**Method:**

A cross-sectional study was conducted by employing 156 glaucoma patients recruited from treatment centers in the Cape Coast Metropolis in Ghana. All the participants underwent dry eye examination and completed the 25-item National Eye Institute Visual Function Questionnaire (NEI-VFQ), the Dry Eye-related Quality of Life Score (DEQS) and the Ocular Surface Disease Index (OSDI). Comparisons of the clinical tests, NEI VFQ-25 subscale item and composite scores and scores of DEQS and OSDI were made among subgroups divided according to the presence of dry eye symptoms or signs. Multivariate logistic regression analysis was performed to investigate the factors that influence DES related-QOL among the patients.

**Results:**

The study involved 156 subjects with a mean age of 47.88 ± 16.0 years and made up of 81 (51.9%) females and 75 (48.1%) males. A One-Way ANOVA was conducted, and the F-statistic (F) indicated that there was a significant difference in the mean scores of the groups. There were significantly lower Tear break-up time (TBUT) values found in the group with definite dry compared with the group without DES and the group that was symptomatic with no signs DES in both eyes, [(F(3,151) = 13.703, p<0.001 (RE): (F(3,152) = 18.992, p<0.001 (LE)]. Similar results were found for Schirmer test (ST) [(F (3,151) = 28.895, p<0.001 (RE): (F (3,152) = 17.410, p<0.001 (LE)]. There was statistically significant difference in the mean composite score (64.93 ± 20.27) for the NEI VFQ-25 and sub-scale score of ocular pain, which was significantly lower in the group with definite dry as compared to other sub-groups (F(3,152) = 4.559, p = 0.004). OSDI scores of the group with definite dry eye (47.69–19.17) and the group that was symptomatic but with no signs (38.90–22.44) were significantly higher than those without dry eye and those that were asymptomatic but had a sign (F(3,152) = 17.896, p<0.001), with a similar trend occurring in the groups with relation to DEQS scores (F(3,152) = 8.775, p<0.001). There was a strong correlation between the DEQS and the OSDI questionnaires, and a weak correlation between the DEQS and the NEI VFQ-25 questionnaire after adjusting for all other factors (all p < 0.01).

**Conclusion:**

The study established a high presence of DES and consequently low DES related-QOL in glaucoma patients. Dry eye questionnaires are able to discriminate those who have definite dry eye from the other groups, showing its appropriateness for clinical use in glaucoma patients. Ocular surface evaluation should be conducted among glaucoma patients on topical anti-glaucoma therapy to ensure the timely detection and treatment of signs and symptoms of DES and improvement of dry-eye related QOL.

## Introduction

Glaucoma is a chronic and blinding condition that is medically and surgically irreversible, but prompt detection and treatment through strict intraocular pressure control can slow the progress of the disease [1, 2]. In Sub-Saharan Africa, where glaucoma is most prevalent, the majority (about 90%) of cases are Primary Open Angle Glaucoma (POAG), and are treated with topical anti-glaucoma drops for an extended time, since surgical uptake is uncommon [3, 4]. Glaucoma drops, however, contain preservatives that are meant to prevent or inhibit microbial growth and extend the shelf life of the drugs, but these ingredients also produce undesired ocular surface changes with long term use [5]. Ocular surface changes, such as DES, have been reported in glaucoma patients on topical medication due to toxic response from the ocular mucosa surface [5–8].

Dry eye is a multifactorial disease of the ocular surface characterized by a loss of homeostasis of the tear film, and accompanied by ocular symptoms, in which tear film instability and hyper- osmolarity, ocular surface inflammation and damage, and neuro- sensory abnormalities play etiological roles [9]. The extended use of topical drugs that contain preservatives such as Benzalkonium Chloride can cause alteration in tear volume and elevated level of tear evaporation which then brings the undesired reaction from the ocular mucosa surface [6]. Ubiquitous body of literature on animal and laboratory studies have observed the effect of preservatives on the conjunctival and corneal cells [10, 11]. Also, non-randomised controlled studies have provided some supporting evidence of the toxicity of Benzalkonium Chloride in ophthalmic patients based on the frequency of drop instillation [6, 8, 12].

Preservatives affect the ocular surface by promoting the expression of inflammatory cell markers [13], damaging the epithelial cells, causing lacrimal cell death through apoptosis, causing changes in the sensory nerves in the cornea and conjunctiva, and reducing the goblet cell density, all culminating in tear hyperosmolarity and tear film instability [8, 14, 15]. The resulting punctate epithelial keratitis and epithelial cell surface damage interfere with the ability of the ocular surface to wet easily, leading to symptoms. The variety of DES symptoms are derived from activation of sensory nerves in the cornea and conjunctiva and it includes feelings of ocular surface discomfort, irritation, dryness, grittiness, burning, itching, and photophobia [16]. Dry eye symptoms are especially worse in glaucoma patients who are usually above 40 years, as they may suffer from the double burden of age-related DES [17, 18]. In view of this, subjectively investigating the impact of dry eye on a glaucoma patient’s daily life, especially for symptoms of discomfort, together with objective clinical measures to initiate treatment is vital in ensuring compliance with glaucoma medications and in the follow-up care. There are a number of diagnostic workup at evaluating patient-reported dry eye symptoms using validated questionnaires, such as the Ocular Surface Disease Index (OSDI) and the Dry Eye-related Quality of Life Score (DEQS) [16]. Clinical signs and evaluation of dry eye include ocular surface staining; evaluation of the cornea and tears for debris, mucus, or keratitis; evaluation of the lids and meibomian glands, tear film break-up time (TBUT), Schirmer Test (ST) score and tear film osmolarity evaluation [19]. The accepted diagnostic criteria requires that one ocular symptom and one ocular sign be satisfied, but better performance, broader characterization of dry eye and vision-related QOL can be achieved when both specific and more general tests are used in parallel or in series [4, 12]. This study, therefore, uses the National Eye Institute Visual Function Questionnaire (NEI VFQ-25), the Dry Eye-related Quality of Life Score (DEQS) and the Ocular Surface Disease Index (OSDI) questionnaire to determine the vision-related QOL of POAG patients as well examine the impact of DES on the QOL of those patients. The results from this study will be useful in making clinical decisions on the need to make ocular surface assessment a routine in the treatment regimen for glaucoma patients.

## Materials and methods

### Study design

A hospital-based, descriptive cross-sectional study was conducted among patients being treated for glaucoma from three referral facilities in the Central Region of Ghana, namely the University of Cape Coast Hospital, Bishop Akon Memorial Christian Eye Centre and the Cape Coast Teaching Hospital, by adhering to the guidelines in the Declaration of Helsinki for research involving human subjects. The study was also approved by the University of Cape Coast Institutional Review Board and all participants provided written informed consent before participating in the study.

### Subjects

A total of 156 glaucoma patients were included in this study. The sample size was determined using the fishers’ formula with glaucoma prevalence at 7.89% [20].

$$\frac{N=Z^{2}\left(1-P\right)P}{w^{2}}$$

Where,

N = sample size

Z = constant = 1.96

P = Prevalence of glaucoma = 7.89%

w = confidence interval = 95%

Therefore, a minimum sample size of 112 was calculated, but this was increased to 156 to cater for attrition and non-responses. Participants were purposively selected consecutively to remove bias. The inclusion criteria were confirmed glaucoma patients aged 18 years and above, had been on topical anti-glaucoma medication (with preservative) for at least 6 months, and best-corrected visual acuity of 0.7logMAR in the worst eye [21]. Exclusion criteria were glaucoma patients with certain systemic conditions that affect the ocular surface (e.g., rheumatoid arthritis, Sjogren’s syndrome) or ocular conditions (egs. infectious diseases, corneal abrasion, pterygium etc), existence of an absolute central visual field defect, ocular surgery in the preceding 6 months of the study, any known allergy or hypersensitivity to the topical anti-glaucoma medication used and patients who were on tear supplements. Also, patients who had been diagnosed with DES, including patients who were being treated for DES, prior to the use of the topical anti-glaucoma therapy as documented in their patient folders or through verbal responses, were excluded. The exclusion criteria was to ensure that the DES in participants was not caused by other factors.

All cases of glaucoma were previously diagnosed by an Ophthalmologist according to the International classification of diseases (ICD-10 or ICD-11) and involved tonometry measures of intraocular pressure (IOP) being more than 21 mmHg on two or more appointments, the presence of glaucomatous optic nerve head (ONH) established by a dilated fundus examination and Optical Coherence Tomography (OCT) scan, and three or more dependable Visual Field Tests (VFT) done at specific intervals on different days with the glaucoma hemifield test (GHT) outside the normal limits. Patients who did not have an IOP of more than 21 mmHg but were diagnosed to have glaucoma based on the presence of a glaucomatous optic nerve head and a glaucoma hemifield test being outside normal limits were included in the study.

### Data collection process

Data collection took place between February and May 2019 by means of structured questionnaires and two clinical diagnostic tests. The diagnostic tests used were TBUT and ST which were performed after external ocular examination of participants [4, 12]. Subjects were engaged in the consulting room after routine check-ups and the entire process explained to them, before versions of the NEI-VFQ 25, OSDI and the DEQS questionnaires were administered to them. The demographic characteristics (age, gender and occupation) were first obtained from the patient’s folder and triangulated with the patients. The data collection process lasted for about 30 minutes on every participant.

#### External examination

External ocular examination of all subjects with the slit-lamp biomicroscope (Haag-Streit technological, BM 900, USA) was carried out to ensure that they did not have other ocular pathologies (e.g. corneal abrasion, pterygium) that would affect the results of the study.

#### Assessment of ocular surface and grading

The set criteria for dry eye disease diagnosis, by TFOS DEWS II Diagnostic Methodology suggests that the simultaneous presence of symptoms and at least one sign is indicative of DES, with patients experiencing at least one symptom often or always is considered as symptomatic [16]. The presence of DES signs was documented by the TBUT, ST and corneal staining [16, 22]. The grading of the ocular surface staining was done by an experienced Optometrist.

The tests were done in the following order: The TBUT was measured first, followed by the assessment of the ocular surface by using corneal staining, and finally the ST was performed. Visual functioning of participants in this study were not considered because the anticipated range of function loss owing to dry eye was slim because all selected participants had a best-corrected visual acuity of 0.7LogMAR in the worst eye.

#### Tear break-up time

To determine TBUT, a fluorescein-impregnated strip was wetted with a drop of normal saline. The procedures for TBUT was performed as previously described where scores less than 10 seconds were considered abnormal and scores greater than 10 seconds were normal [23, 24].

#### Corneal staining

Corneal staining was defined as having more than one dot of fluorescein staining over the surface of the cornea and was assessed using the National Eye Institute grading system. The degree of corneal staining was graded on a 4-point scale (0–3) [25]. The scores obtained in the 5 regions of the cornea (superior, inferior, temporal, nasal, and central) were added to get a total corneal staining grade for each eye, which ranged from 0 to 15.

#### Schirmer test I

The Schirmer test I (ST) was carried out with filter paper strips placed at the meeting point between the temporal third of the lower eyelid. The test was performed without topical anaesthesia which means both basal tear secretion as well as reflex tears were measured. The wetted length was measured after 5 minutes. A score of 10mm/5min and below was considered clinically significant tear deficiency [26].

#### Ocular surface disease index questionnaire

The 12-item OSDI questionnaire assess the symptoms of ocular irritation in dry eye disease and how they affect functioning related to vision. The questionnaire was administered to all participants by a masked Optometrist. The Optometrist was masked to prevent the unconscious conveying of information or exhibiting behaviors that could influence the participant’s answers. Individual OSDI questions were scored on a 5-point Likert scale, with scores of 0, 1, 2, 3, and 4 corresponding to answers of none of the time, some of the time, half of the time, most of the time, and all of the time, respectively. A total score was calculated using the following equation: 25 x [(sum of scores for all individual questions)/(total number of questions answered)], obtaining a total score ranging from 0 to 100. Scores of 0–12 being normal, 13–22 being mild, 23–32 being moderate, and 33–100 having severe symptoms [27–29].

#### National Eye Institute Visual Function Questionnaire-25

The National Eye Institute Visual Function Questionnaire-25(NEI-VFQ-25) assesses the effect of visual impairment on the patient’s current health-related QoL, including questions dealing with irritation in and around the eyes. It consists of a base set of 25- vision targeted questions to generate the respondent’s global vision rating, difficulty with near vision and distance vision activities, limitations to functions and roles, dependency on others, mental health symptoms, driving difficulties, limitations with peripheral and color vision, and ocular pain. The NEI-VFQ scores range from 0 to 100 with lower scores indicating more problems or symptoms. The composite score is the overall score of the VFQ-25, simply averaging the vision-targeted subscale scores, excluding the general health rating question [30].

#### Dry Eye-Related Quality-of-Life Score (DEQS)

Dry Eye-Related Quality-of-Life Score (DEQS) questionnaire is used to diagnose dry eye and quantify its severity level. It was administered to elicit respondent’s responses to various dry eye symptoms and their influence on day-to-day vision-related QOL. The DEQS consists of 12 questions that measure various aspects of a person’s life affected by dry eye. Each question is rated on a five-point scale, with scores ranging from 0 (no impact) to 4 (severe impact). The total score is calculated by summing the scores for each question, with a maximum score of 48. Higher scores indicate a greater impact of dry eye on QOL. The score categorization was similar to the one used for the OSDI questionnaire [31].

### Statistical analysis

Definite dry eye was defined as the simultaneous presence of symptoms (OSDI ≥ 13) and at least one sign (TBUT ≤ 10 sec or ST ≤ 10mm/5min or positive staining). Subjects were considered symptomatic when at least one of the symptoms mentioned in the OSDI questionnaire was experienced often or all the time [32]. Clinical tests were considered indicative of signs in the following instances: ST score ≤ 10 mm or TBUT ≤ 10 seconds in at least one eye. To assess the impact of DES, patients were divided into four groups, according to their dry eye status and the presence of signs and symptoms of DES: Group A (Patients with no DES), Group B (Patients with definite DES), Group C (Asymptomatic patients with a sign of dry eye) and Group D (Symptomatic patients with no signs of dry eye).

Descriptive statistics was performed and mean scores with standard deviations were compared for TBUT, ST, OSDI, DEQS and each NEI-VFQ-25 sub-scale and composite scores. Binary logistic regression was done to find factors associated with the outcome variables. The variables that were discovered to be associated at a p-value ≤ 0.05 were then entered into the multiple logistic regressions to investigate the relationship between the clinical variables and the VFQ-25 scores. The odds ratio with 95% confidence interval at p ≤ 0.05 was used to determine a statistically significant association. The data was analysed using SPSS for MacOS, version 23.0; SPSS, Inc., Chicago, IL.

## Results

### Demographic and clinical characteristics

The demographic characteristics of participants are shown in Table 1. A total of 156 patients with glaucoma, who were all black Africans participated in the study. Their ages ranged from 18 to 84 years with a mean age of 47.88 ± 16.0 years. Slightly more than half, 81 (51.9%) were females and a high proportion of them 69 (44.2%) were 50 years and above. In terms of glaucoma drops, the majority 89(57.1%), were using beta-blockers, mostly Timolol.

**Table 1:** General characteristics of study population.

Variables	Male(n = 75)	Female(n = 81)	Total (n = 156)
**Age(years)**			
18–34	14(18.7%)	19(23.5%)	33(21.2%)
35–49	25(33.3%)	26(32.1%)	51(32.7%)
50–64	23(30.7%)	24(29.6%)	47(30.1%)
65–80	11(14.7%)	11(13.6%)	22(14.1%)
>80	2 (2.7%)	1 (1.2%)	3(1.9%)
**Dry Eye Syndrome Status (TBUT)**			
Normal (<10 seconds)	22(29.3%)	23(28.4%)	45(28.2%)
Abnormal (>10 seconds)	53(70.7%)	58(71.6%)	111 (71.8%)
**Tear Status (Schirmer test)**			
Sufficient	27(36.0%)	38(46.9%)	65(41.7%)
Insufficient	48(64.0%)	43(53.1%)	91(58.3%)
**Type of anti-glaucoma used by participant**			
Beta—blockers	46(61.3%)	43(53.1%)	89(57.1%)
Prostaglandin analogue	11(14.7%)	18(22.2%)	29(18.6%)
Combination Therapy	18(24.0%)	20(22.7%)	38(24.3%)
**Specific anti-glaucoma drug used by participants**			
Timolol	46(61.3%)	43(53.1%)	89(57.1%)
Latanoprost	11(14.7%)	18(22.2%)	29(18.6%)
Gamfort	7(9.3%)	10(12.34%)	17(10.8%)
Lumigan	5(6.7%)	1(1.2%)	6(3.9%)
Combigan	6(8%)	9(11.1%)	15(9.6%)

Tear break-up time assessment indicated that, the majority 111 (71.8%) had a score less than 10 sec, and their tear film were considered abnormal, whereas 45 (28.2%) had a score greater than 10 sec, and the tear film were considered as normal. As indicated in Table 2, only a few 10 (6.4%) of the subjects had no DES (Group A), with the majority 96 (61.5%) diagnosed with definite DES (group B), 21(13.5%) were asymptomatic patients but had a sign (Group C), and 29(18.6%) patients were symptomatic but had no signs (Group D). There was a statistically significant differences between the mean TBUT values of both eyes among the four sub-groups as determined by a one-way ANOVA, with significantly lower values of TBUT found in group B compared with group A and group D in both eyes, [(F(3,151) = 13.703, *p*<0.001 (RE): (F(3,152) = 18.992, *p*<0.001 (LE)]. The comparisons on the ST showed similar results [(F(3,151) = 28.895, P<0.001 (LE), (F(3,152) = 17.410, *p*<0.001 (LE)].

**Table 2:** Group characteristics of participants.

Characteristics	Total (n = 156)	Patients with no DES (n = 10)	Patients with definite DES (n = 96)	Asymptomatic patients with a sign of DES (n = 21)	Symptomatic patients with no signs of DES (n = 29)
**Gender**					
Male	75 (48.1%)	6(60.0%)	45(46.9%)	11(52.4%)	13(44.9%)
Female	81 (51.9%)	4 (40%)	51(53.1%)	10(47.6%)	16(55.1%)
**Age**					
Average(years)	47.88±16.0	40.40±11.1	49.35±15.8	48.24±4	47.88±17.5
Range	18–84	21–54	18–84	19–81	18–75
**Education**					
No education	10(6.5%)	0(0.0%)	5(5.3%)	2(9.5%)	3(10.3%)
Primary	13(8.4%)	1(10.0)	7(7.4%)	2(9.5%)	3(10.3%)
Secondary	105(67.7%)	8(80.0%)	67(70.5%)	11(52.4%)	19(65.5%)
Tertiary	27(17.4%)	1(10.0%)	16(16.8%)	6(28.6%)	4(13.8%)
**TBUT of subgroups (seconds)**					
Right eye		9.80 ± 3.05	6.58 ± 2.67	7.43 ± 2.94	9.93 ± 2.62
Left eye		9.80 ± 2.35	6.69 ± 2.66	7.57 ± 2.88	10.69 ± 2.58
**ST of four subgroups (mm)**					
Right eye		18.70 ± 9.37	8.76 ± 6.07	10.38 ± 5.79	21.07 ±8.12
Left eye		17.50 ± 8.70	9.77 ± 7.41	11.14 ± 6.90	20.90 ± 8.495

### Mean scores for each sub-scale and composite score for VFQ-25

The mean scores and standard deviation of each NEI VFQ-25 sub-scale are shown in Table 3, ranging from 57.12 for general vision to 77.28 for social function. The mean composite score was 64.93 ± 20.27 with the following scoring below the composite score; General vision, 57.12±28.76; general health, 58.01±27.53; near activity, 59.87±28.19; mental health, 63.00±21.94; and peripheral vision, 63.97±28.37. The analysis of the composite and each sub-scale score among the four subgroups showed a statistically significant difference in the composite score and only the scores of ocular pain, which was significantly lower in group B as compared to groups A, C and D C, as shown in Table 3 (F(3,152) = 4.559, p = 0.004). The other subscale scores of the NEI- VFQ-25 showed no significant difference within the four groups.

**Table 3:** The mean scores ± SD and the analysis of the composite score and each subscale score of the NEI VFQ-25 among the four sub-groups.

	Patients with no DES (n = 10)	Patients with definite DES (n = 96)	Asymptomatic patients with a sign of DES (n = 21)	Symptomatic patients with no signs of DES (n = 29)	Average±SD	F	*p*
General Health	65.00–24.15	56.50–27.913	61.21–28.81	58.01–27.53	58.01±27.53	0.444	0.722
General Vision	64.00–22.71	53.75–27.84	59.52–32.32	64.14–30.42	57.12±28.76	1.261	0.290
Ocular Pain	71.20–20.92	56.64–21.40	69.71–20.78	69.66–24.42	61.75±22.62	4.559	0.004*
Near activity	64.20–31.55	57.43–27.46	62.10–29.31	64.83–29.20	59.87±28.19	0.657	0.580
Distance activity	71.20–29.40	62.40–28.00	72.24–29.90	70.98–25.10	65.88±27.88	1.316	0.271
Social function	69.00–30.43	75.15–24.58	72.90–29.50	78.29–25.14	75.01±25.60	0.380	0.768
Mental health	71.70–18.73	62.70–21.42	60.95–25.02	62.48–22.78	63.00±21.94	0.592	0.621
Role difficulties	71.50–16.41	63.65–28.37	70.48–30.07	68.97–25.39	66.06±27.42	0.665	0.158
Dependency	73.70–18.54	69.81–24.81	82.29–23.34	76.66–26.12	73.01±24.73	1.758	0.158
Color vision	70.00–30.73	74.79–27.29	86.90–23.21	81.03–21.81	77.28±26.25	1.706	0.168
Peripheral vision	55.00–32.91	62.58–27.34	72.38–29.30	65.52–29.44	63.97±28.37	1.055	0.370
Composite score	63.90–20.50	62.82–19.30	69.71–23.10	68.79–21.15	64.93±20.27	5.067	0.003
Driving Score	68.50–27.70	71.89–20.73	78.50–12.01	78.00–16.79	73.35±19.79	0.425	0.736

*p-value < 0.05.

The F is the F-statistic, calculated by dividing the mean square between the groups by the mean square within the groups.

### Comparison of NEI-VFQ-25 scores to OSDI and DEQS scores

As indicated in Table 4, the NEI-VFQ-25 values for pain were significantly higher in groups A, C and D than those of group B (F(3,152) = 4.559, P = 0.004). Comparatively, OSDI scores of Groups B (47.69–19.17) and D (38.90–22.44) were significantly higher than those of Groups A and C (F(3,152) = 17.896, p<0.001), with a similar trend occurring in the groups with relation to DEQS scores (F(3,152) = 8.775, p<0.001). For the clinical measurements, ST values in the right eye were significantly lower in both Groups B and C compared to Groups A and D (F(3,152) = 24.176, p<0.001), as was the case for ST values in the left eye(F(3,152) = 14.412, p<0.001) of Groups B and D which were significantly higher than those of Groups A and C (F = 17.896, p<0.001). Also, significantly lower values of TBUT were found in Groups B and C as compared to Group A and D for both the right eye (F(3,152) = 21.937, p<0.001) and the left eye (F(3,152) = 26.656, p<0.001). QOL status as measured by OSDI and DEQS among the groups were statistically significantly different (p<0001) and were predictive of each other. There was a strong and significant correlation between the OSDI and DEQS questionnaires: r (156) = 0.604, p < .001. There was, however, a weak correlation between composite score of NEI-VFQ-25 questionnaire with the OSDI and the DEQS: r (156) = -0.234, p = 0.003, r (156) = -0.217, p = 0.0071 even after the adjustment of all other factors.

**Table 4:** Comparison of VFQ-25, OSDI, DEQS, ST and TBUT among the sub-groups.

	Patients with no DES (n = 10)	Patients with definite DES(n = 96)	Asymptomatic patients with a sign of DES(n = 21)	Symptomatic patients with no signs of DES(n = 29)	F-statistic	p
**NEI-VFQ** Composite Score	63.90±20.50	62.82±10.21	69.71±23.09	68.79±21.15	1.098	0.352
**NEI-VFQ** Ocular Pain	71.20±20.92	56.64±21.40	69.71±20.80	69.66±24.42	4.559	0.004*
**OSDI**	16.40–10.21	47.69–19.17	19.52–18.58	38.90–22.44	17.896	0.000*
**DEQS**	21.10–17.05	43.57–17.23	25.48–20.13	42.83–23.55	8.775	0.000*
**ST (mm)**						
Right eye	18.50–9.24	8.72–5.99	7.86–3.60	18.10–6.88	24.176	0.000*
Left eye	17.50–8.70	9.77–7.41	8.0–3.60	18.03–7.01	14.412	0.000*
**TBUT (seconds)**						
Right eye	10.80–1.48	6.58–2.68	7.43–2.94	10.55–2.50	21.937	0.000*
Left eye	11.10–2.51	6.70–2.64	7.57–2.87	11.21–2.56	26.656	0.000*

*p-value < 0.*05*.

### Factors that influence dry eye syndrome-related quality of life in glaucoma patients

A multiple logistic regressions analysis was done to investigate factors that influence DES related QOL among the patients. The result, as shown in Table 5 reveal that severity of symptoms, as measured by higher OSDI and DEQS scores were associated with higher Odds for developing DES after adjusting for age. Patients using combination therapy, (beta blockers with prostaglandin analogues and beta blockers with alpha-2 agonist) were less likely to develop DES as compared to patients using only beta blockers.

**Table 5:** Factors associated with presence of DES amongst glaucoma patients.

Variables	DES Status		COR (95% CI)	AOR (95% CI)
	Yes	No		
**Sex**				
Male	53	22	1.00	1.00
Female	58	23	1.047(0.523,2.093)	1.068(0.490,2.33)
**Age Category**				
18–34	22	11	1.00	1.00
35–49	35	16	1.094(0.430,2.785)	0.983(0.347,2.779)
50–64	36	11	1.636(0.608,4.403)	1.651(0.532,5.121)
65–80	16	6	1.333(0.408,4.361)	1.232(0.30,5.063)
>80	2	1	1.00(0.081,12.270)	0.851(0.047,15.482)
**OSDI Grading**				
Normal	9	6	1.00	1.00
Mild	17	11	0.485(0.154,1.522)	0.668(0.135,3.297)
Moderate	20	7	0.499(0.202,1.233)	0.661(0.098,4.436)
Severe	65	21	0.923(0.343,2.488)	1.116(0.181,6.895)
**DEQS Grading**				
Normal	7	9	1.00	1.00
Mild	14	6	3.000 (0.759,11.8649)	4.346(0.783,24.130)
Moderate	22	6	4.714 (1.237,17.970)	4.072(0.622,26.660)
Severe	68	24	3.643 (1.223,10.855)	2.991(0.521,17.187)
**Class of Anti-Glaucoma Medication**				
Prostaglandin Analogue	29	9	1.00	1.00
Beta Blocker+Prostaglandin Analogue	9	8	0.853(0.343,2.120)	0.273(0.073,1.022)*
Beta Blocker+Alpha-2 Agonist	5	10	0.298(0.101,0.881)	0.159 (0.038,0.662)*
Beta Blocker	68	18	0.132 (0.040, 0.436)	1.329 (0.508,3.483)
NEI-VFQ Compposite Score	64.23–19.98	66.67–21.10	0.994(0.977,1.011)	0.995 (0.973,1.018)

*p-value < 0.05.

COR = crude Odds ratio.

AOR = Adjusted Odds ratio.

## Discussion

The use of topical anti-glaucoma medications is the most common and affordable in low resource environments [33]. Regrettably, the prolong use of these IOP lowering medications is linked to symptoms of toxicity from preservatives in these topical medications, which cause ocular inflammation, injuries to the corneal and conjunctiva epithelial cells, allergies and the development DES. Dry eye syndrome has been associated with self-reported impairment in the execution of many daily tasks including reading, using a computer, watching television, and driving [34]. However, limited research has been conducted to determine the comparative effect of DES in glaucoma patients using different questionnaires and objective methods. The present study, which used different approaches to measure vision-related QOL, was done to establish the presence of DES in patients being managed with different topical anti-glaucoma medications and to examine the impact of DES on their QOL.

In Ghana, population surveys conducted indicate that the country has the highest prevalence of glaucoma in Africa, and third in the world after Barbados and St. Lucia [35, 36]. The prevalence of glaucoma in Ghana ranges from 6.5% to 8.5% among those 40 years and above, and was found to be the second major cause of blindness accounting for 19.4%, after cataract (54.8%) [37]. Though there is no clear gender predilection for glaucoma in this study, there were slightly more females than males among the glaucoma patients recruited. Glaucoma is a chronic condition, so the slight disparity in gender could be attributed to the fact that, females are more likely to be concerned with their eye health and therefore, attend reviews more than their male counterparts [38]. The prevalence of dry eye symptoms in the study population according to the OSDI was 80.1% (61.5% had definite DES) which was greater than the reported prevalence ranging from 14.4% to 33% in the general population [16, 18, 32] and was proportionally more in the aged and females. These results are consistent with previous studies [39, 40] which reported that comorbid factors such as aging was associated with evaporative dry eye and tear film instability and suggested that females were more likely to have DES than men due to reduced androgen levels.

Though the different classes of topical anti-glaucoma therapy have a singular aim, their regimen differs. In this study, paticipants who used beta-blockers and beta-blockers + alpha-2 agonists instilled their medication twice daily at a 12 hour interval. Patients who used prostaglandin analogues and beta-blockers + prostaglandin analogues instilled their medication once daily at evenings. This study found that the type of drug used by the patients was a factor for experiencing DES, with patients using Beta-blockers having the highest Odds for DES. A previous report [41] suggests that the antagonistic activity of Beta-blockers towards the sympathetic nervous system could lead to dryness in the eye. Indeed, the majority of glaucoma medications used in Ghana are Beta-blockers which contain preservatives [33]. Compared to participants on beta-blockers, participants on combination therapy had lower odds for DES, corroborating a previous study that suggested that combination therapy is typically formulated to complement each other and minimize side effects, including dry eye [42]. Prostaglandin analogues, which may be preservative-free, are more expensive and are least opted for by patients in low resourced environments because of the financial burden it will place on them [33]. For this reason, it was not surprising that nearly all the patients in the present study were on preserved topical therapy containining benzalkonium chloride as a preservative. Preservatives such as benzalkonium chloride, chlorobutanol and methylparaben contained in these medications can cause surface epithelial cell damage and punctuate epithelial keratitis [15]. The resultant mucosa surface defects reduce tear secretion, causing dryness of the eye in glaucoma patients. In contrast, other studies [8, 43–46] have shown that the use of only preservative free eye drops drastically reduces the signs of ocular surface alteration in glaucoma patients, with other forms of preservation methods also reducing the side effects on the ocular surface in comparison to drugs preserved with high levels of Benzalkonium Chloride.

Some studies have shown that patients suffering from symptoms of dry eye disease have a lower QOL and reported negative effects on visual function, their ability to carry out daily activities and work productivity [2, 47]. Consistent with those studies, the findings in this study indicate that, the composite score for NEI VFQ and sub-scale for ocular pain score were significantly lower and related to dry eye in participants with definite dry eye or signs or symptoms of dry eye (Group B, C and D), compared to participants without dry eye (Group A). This shows that DES can have a significantly negative impact on the overall vision-related QOL of glaucoma patients who are on topical anti-glaucoma medication. In this study, the subscale score for ocular pain ranged from 68.5 to 89.5 in patients with definite dry eye (Group B), showing that dry eye may contribute to increased ocular pain and discomfort [21, 48]. It is believed that the adverse effect on the QOL is partially contributed to by ocular pain [47]. It can also be noted that the symptomatic patients who did not show signs of dry eye and asymptomatic subjects with a dry eye sign reported similar scores for both the composite score and ocular pain score for the NEI-VFQ, consistent with the findings of a study in the general population in China [32]. The findings show that unpleasant symptoms of dry eye, such as ocular grittiness, burning or stinging, blurred vision, foreign body sensation and photophobia as well as signs of DES can affect QOL of patients negatively.

Previous studies have shown a link between reported symptoms by patients and clinically measured parameters [32, 49, 50]. While some clinicians rely heavily on reported symptoms for diagnosing DES in glaucoma patients, it is generally agreed that the earlier onset of dry eye disease is related to aqueous deficiency and tear film stability [12, 13]. Hence, clinical measures of the aqueous tear production and tear evaporation are recommended to be performed routinely by clinicians to complement symptomatic assessment [16]. This study also found that subjective symptom measurements reflected the objective clinical measurements, which revealed that the values obtained for TBUT and ST were significantly lower in participants with definite dry eye and asymptomatic patients with a sign of DES as compared to patients without dry eye and patients with symptoms but no signs. The results show that some symptomatic patients may not present with clinical signs, and may be missed out if clinical measures are only used. Subjective symptom assessment should therefore be a critical outcome measure when assessing patients for improvement in their QOL [51].

One of the objectives for this study was to compare the relative measures of dry eye using different symptom questionnaires to gain better understanding of the patients status. The OSDI is a disease-specific measure that surveys the ocular symptoms, vision-related function and environmental triggers associated with dry eye and the NEI VFQ-25 is a vision-specific method which is more suitable to measure the overall impact of a chronic ocular disease on vision-related QOL. More specifically, the OSDI is used to evaluate how much the symptoms of dry eye affect the person in the past one week, while NEI-VFQ incorporates questions addressing both the frequency and intensity of symptoms and their impact on activities with no specified recall period, and thus, it is more appropriate for assessing the total effect of a chronic ocular disease on the QOL of a patient [52]. With the OSDI and the NEI-VFQ measuring slightly different things in different time ranges, it was imperative to use the Dry Eye-Related Quality-of-Life score (DEQS) to have a disease specific QOL measure. The DEQS elicited respondents’ knowledge on various dry eye symptoms and their influence on day-to-day vision-related QOL. The combination of the generic and disease specific questionnaires gave a more clear picture of vision-related QOL than used in previous studies. This study found strong correlations between the DEQS and the OSDI questionnaires but a weak correlation between the DEQS and the NEI VFQ-25 questionnaire. The strong correlations imply that, the DEQS questionnaire could predict the outcome of the OSDI and vice versa. As shown in Tables 3 and 4, the NEI-VFQ-25 questionnaire was able to discriminate those who had definite dry eye from the other groups, with the value for sub-scale for ocular pain significantly lower than the other groups. The results were associated with the findings from OSDI and DEQS questionnaires, which revealed higher scores for subjects with definite dry eye and those that were symptomatic but had no signs of dry eye compared to those subjects without dry and those that were asymptomatic but had a sign of dry eye. This result is consistent with the finding that even though the questionnaires differ, they all have adequate measurement precision and show psychometric validity for clinical use in African glaucoma patients [53, 54].

Overall, the study reveals that symptoms of DES can impact on the QOL of glaucoma patients, independent of the progression and the severity of the glaucoma which this study did not measure. This is of particular clinical importance because glaucoma patients encounter anxiety concerning the possibility of increased disability due to the disease, and will now have to also equally worry about increased symptoms due to DES, and reduction of QOL. Thus, the ocular surface integrity, especially of patients already diagnosed with DES should be assessed frequently when they have been prescribed topical anti-glaucoma medication, to guarantee the timely discovery and management of adverse reactions to the medication on the ocular surface.

While this study was of clinical significance, it was limited by a number of factors. First, the study did not measure how long the patients had used glaucoma medication and the severity of their condition to relate these to their symptoms and QOL. Scores categorization was done based on previous studies, and not the use of advanced psychometric analysis like the Rasch analysis. Further, the duration of use, dose frequency and compliance to anti-glaucoma drugs by subjects were not recorded due to lack of information. It may also be possible that some patients had used other unreported medication or may have undiagnosed systemic conditions like diabetes which may have negatively impacted on the results. The study also excluded patients who had a history of DES prior to the use of topical anti-glaucoma therapy, and also excluded participants who were being treated for DES already. This was done so as not to confound the results, but may have led to the underestimation of the proportion of patients with DES. Again, while the subjects were divided into subgroups, this study did not use a randomised controlled group to match the outcome of the study. Still concerning the subgroups, multiple group comparisons were not accounted for in the sample size calculation as the groups and their members were determined only after the diagnostics tests. Despite these limitaions, the study has revealing findings that have significant implications for clinical care of glaucoma patients in our population due to the overrelaience on topical therapy for glaucoma treatment.

## Conclusions

The results of this study indicate that the signs and symptoms of DES are related to a negative effect on vision-related QOL among glaucoma patients on topical, chronic anti-glaucoma medication. This is mainly represented as ocular pain and discomfort which were collaborated by clinical measures and symptom scores from different validated questionnaires. While clinical examination findings remain fundamental for the evaluation of dry eye, it is also equally essential to pay attention to the subjective symptoms and QOL scores in devising an appropriate treatment regimen. There is the need to conduct large-scale randomised controlled trials that would compare the toxic effects of specific medications and the impact on glaucoma patients’ QOL.

## Supporting information

S1 File (SAV)
